# Laparoscopically guided transversus abdominis plane block versus local wound analgesia in laparoscopic surgery for peritoneal endometriosis: study protocol for a prospective randomized controlled double-blinded LTAP-trial

**DOI:** 10.1186/s13063-022-06004-6

**Published:** 2022-01-18

**Authors:** Anna Terho, Terhi Puhto, Johanna Laru, Outi Uimari, Pasi Ohtonen, Tero Rautio, Sari Koivurova

**Affiliations:** 1 Department of Obstetrics and Gynecology, Oulu University Hospital and PEDEGO Research Unit & Medical Research Center, University of Oulu, PL 23, 90029 OYS Oulu, Finland; 2 Oulu University Hospital and Research unit of Surgery, Anesthesia and Intensive Care, University of Oulu, PL 21, 90029 OYS Oulu, Finland

**Keywords:** Transversus abdominis plane block, LTAP, Laparoscopic gynecologic surgery, Peritoneal endometriosis, Postoperative pain, ERAS, Enhanced recovery after surgery

## Abstract

**Background:**

Ultrasound-guided transversus abdominis plane block (TAP) performed by anesthesiologist has been shown to be an effective and safe analgesia method in abdominal surgery, reducing postoperative opioid consumption. Recently, there has been growing interest to insert TAP under laparoscopic vision (LTAP) by surgeon. LTAP has been used in laparoscopic gastrointestinal surgery, but studies on LTAP in gynecologic laparoscopic surgery are sparse and inconsistent. The purpose of this study is to compare the efficacy of LTAP and local wound analgesia in laparoscopic surgery due to suspected or diagnosed superficial peritoneal endometriosis.

**Methods:**

The LTAP-trial is a prospective randomized controlled double-blinded study comparing the efficacy and safety of LTAP with local wound analgesia in laparoscopic endometriosis surgery. Patients are randomized to receive LTAP with levobupivacaine and wound infiltration with placebo or wound infiltration with levobupivacaine and LTAP with placebo. The primary outcome is postoperative opioid consumption measured by patient-controlled analgesia (PCA) pump. Secondly, subjective postoperative pain up to 24 h postoperatively will be measured by Numeric Rating Scale (NRS). Additional outcome measures are factors related to recovery and length of stay in the hospital as well as a 6-month follow-up survey regarding pain (NRS) and endometriosis-related wellbeing (endometriosis-related health profile, EHP-30) after surgery. A total of 46 patients will be randomized in a proportion of 1:1.

**Discussion:**

Patients with peritoneal endometriosis are often prone to severe postoperative pain that may prohibit their enhanced recovery after laparoscopy. Thus, there is a need for effective postoperative pain management with minimal side-effects. This study focusing on laparoscopically inserted transversus abdominis plane block may provide new insight in dealing with postoperative pain after laparoscopic endometriosis surgery as well as after other gynecologic surgery.

**Trial registration:**

The LTAP-trial -protocol has been prospectively registered to ClinicalTrials.gov, ID: NCT04735770. Registered on February 2021.

## Administrative information

Note: the numbers in curly brackets in this protocol refer to SPIRIT checklist item numbers. The order of the items has been modified to group similar items (see http://www.equator-network.org/reporting-guidelines/spirit-2013-statement-defining-standard-protocol-items-for-clinical-trials/).

**Table:** 

Title {1}	Laparoscopically guided transversus abdominis plane block versus local wound analgesia in laparoscopic peritoneal endometriosis surgery: a prospective, randomized, double-blinded LTAP-trial
Trial registration {2a and 2b}.	ClinicalTrials.gov, ID: NCT04735770. Prospectively registered, February 2021. European Union Drug Regulating Authorities Clinical Trials Database: Eudra-CT 2020-004353-80. Registered 2020.
Protocol version {3}	March 9, 2021, version 1.
Funding {4}	No external funding.
Author details {5a}	MD, Anna Terho, Department of Obstetrics and Gynecology, Oulu University Hospital and PEDEGO Research Unit & Medical Research Center, University of Oulu, Oulu, Finland MD, Terhi Puhto, Oulu University Hospital and Research unit of Surgery, Anesthesia and Intensive care, University of Oulu, PL 21, 90029 OYS, Finland MD, Johanna Laru, Department of Obstetrics and Gynecology, Oulu University Hospital and PEDEGO Research Unit & Medical Research Center, University of Oulu, Oulu, Finland MD, PhD, Outi Uimari, Department of Obstetrics and Gynecology, Oulu University Hospital and PEDEGO Research Unit & Medical Research Center, University of Oulu, Oulu, Finland Pasi Ohtonen, Division of Operative Care, Oulu University Hospital and Research unit of Surgery, Anesthesia and Intensive care, University of Oulu, PL 21, 90029 OYS, Finland Professor (a recent nomination), Tero Rautio, Oulu University Hospital and Research unit of Surgery, Anesthesia and Intensive care, University of Oulu, PL 21, 90029 OYS, Finland MD, PhD, Sari Koivurova, Department of Obstetrics and Gynecology, Oulu University Hospital and PEDEGO Research Unit & Medical Research Center, University of Oulu, Oulu, Finland
Name and contact information for the trial sponsor {5b}	Oulu University Hospital and University of Oulu. Principal Investigator Dr. Sari Koivurova, M.D., Ph.D. Address: PL 24, 90029 OYS, Oulu, Finland Email: sari.koivurova@fimnet.fi Tel: +358 8 3153082
Role of sponsor {5c}	This is a researcher-driven study carried out in Oulu University Hospital, with no outside sponsor or funding. The Principal Investigator is actively involved in planning and executing of the study.

## Introduction

### Background and rationale {6a}

Endometriosis is a chronic disease affecting about 10% of women during their fertile years. Patients often suffer from chronic pelvic pain that decreases markedly their quality of life. Conservative hormonal medication, being the primary treatment option, is often insufficient due to persisting pain, contraindications, or side-effects such as irregular bleeding. Hence, there is also need for surgical treatment of peritoneal endometriosis [1]. Clinical observations have shown that patients with endometriosis tend to suffer from more severe postoperative pain after pelvic surgery than other patients [2]. This may be due to hypersensitization of sensory pelvic nerves, which is caused by the inflammatory nature of endometriosis. Dysmenorrhea, which is the leading symptom of endometriosis, is a prognostic factor for the severity of postoperative pain [3]. The use of epidural or local wound analgesia after endometriosis surgery has been common practice. However, local wound analgesia is often insufficient for endometriosis patients, who tend to suffer from more severe postoperative pain [2]. Epidural analgesia carries a risk of potentially harmful complications and may also prohibit enhanced recovery after surgery (ERAS) and prolong time to discharge. In addition, postoperative opioid consumption also predisposes to complications such as nausea, vomiting, and respiratory depression.

Dorsally inserted transversus abdominis plane block (TAP) has been used as a part of multimodal analgesia in abdominal surgery since the beginning of twenty-first century [4]. TAP blocks the sensory nerves of the abdominal wall unilaterally between the costal margin and the inguinal ligament (T6–L1), and it provides analgesia to the parietal peritoneum, muscles, and abdominal skin accordingly [5]. When inserted in a blind fashion, it carries a risk of visceral injury. To avoid complications, ultrasound-guided transversus abdominis plane block technique (UTAP) was adopted by anesthesiologists. Both methods have been shown to serve as an effective postoperative analgesia reducing postoperative opioid consumption and opioid-related side effects after open and laparoscopic abdominal surgery [6].

Laparoscopically inserted transversus abdominis plane block (LTAP) is a novel technique that has been used by gastrointestinal surgeons, especially in laparoscopic cholecystectomies and colorectal surgery [7, 8] with equally good results in comparison to UTAP [9]. According to a recent meta-analysis, LTAP is safe and superior to local wound analgesia in adults undergoing minimally invasive surgery (such as laparoscopic or robotic abdominal procedures) regarding early pain control, opioid consumption, and patient satisfaction [10]. In another study, a reduction in postoperative pain score measured by visual analog scale was noted even at 1 week after laparoscopic cholecystectomy with LTAP in comparison to local wound analgesia [11]. LTAP has been suggested to reduce postoperative pain and opioid need in gynecologic and endometriosis laparoscopic surgery, but the results of previously published studies have been inconsistent [12–14]. The purpose of this study is to investigate whether LTAP provides a post-laparoscopic opioid-sparing effect in comparison to local wound analgesia, thus diminishing opioid related side-effects and improving ERAS.

### Objectives {7}

The objective of this prospective, randomized, controlled, double-blinded study is to examine whether LTAP is superior, i.e., reduces the postoperative opioid consumption compared to local wound analgesia in treating post-laparoscopic pain in patients suffering from pelvic pain because of suspected endometriosis.

## Methods/design

### Trial design {8}

This is a prospective, randomized, controlled, double-blinded clinical study evaluating the effectiveness and safety of LTAP compared to local wound analgesia in laparoscopic gynecological surgery for superficial peritoneal endometriosis. Randomization at patient level is done with an allocation ratio of 1:1. The active LTAP group will receive a long-acting local anesthetic, levobupivacaine, administered at LTAP points and saline infiltrated at the sites of laparoscopic ports. The control group will receive saline at LTAP points and levobupivacaine at the sites of laparoscopic ports. The patient and the surgeon remain blinded to the allocated group until the end of the study data collection.

**Fig. 1:**
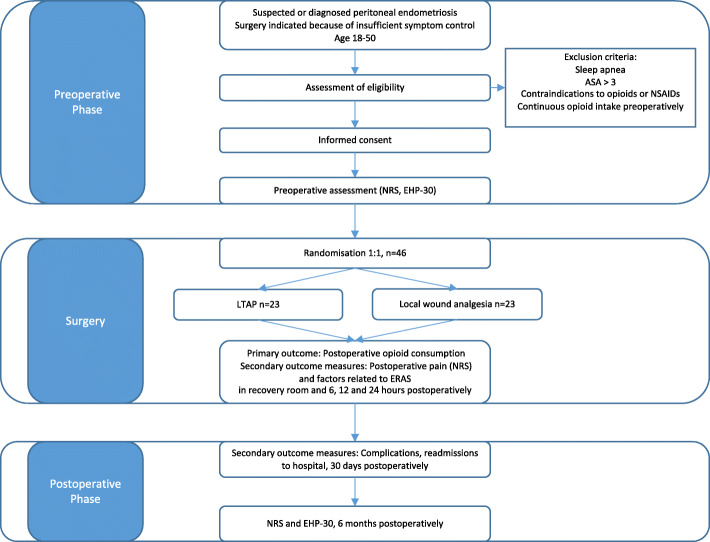
Flow chart describing the study protocol

**Fig. 2:**
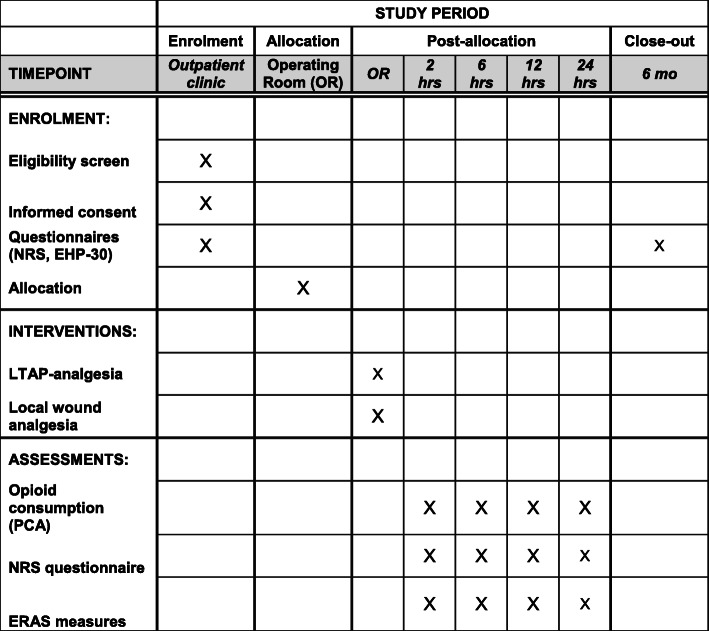
Time of collection of outcomes

The aim of this study is to explore whether LTAP is superior, i.e., offers opioid-sparing effect when compared to local wound analgesia in postoperative pain after laparoscopic gynecological surgery for peritoneal endometriosis. The power calculation for this study was based on 50% reduction in the consumption of opioids during the postoperative period, which we estimated to be clinically relevant. The study protocol flow chart and time of collection of outcomes are described in Figs. 1 and 2, respectively.

### Study setting {9}

This is a single-center trial located in a tertiary university hospital in Finland.

### Eligibility criteria {10}

The inclusion criteria are as follows:

- Age 18–50 years
- Diagnosed or suspected peritoneal endometriosis and laparoscopic surgery indicated
- ASA 1–3

The exclusion criteria:

- Sleep apnea
- ASA > 3
- Contraindications to opioids or non-steroidal anti-inflammatory drugs
- Continuous opioid intake preoperatively

The laparoscopies and LTAP administration will be performed by two experienced laparoscopists, who work within a multi-professional team focusing on endometriosis surgery in a tertiary hospital setting.

### Who will take informed consent? {26a}

Informed consent will be obtained during the preoperative clinical review at the outpatient clinic by the operating gynecologist.

### Additional consent provisions for collection and use of participant data and biological specimens {26b}

Not applicable. No biological specimens will be collected.

## Interventions

### Explanation for the choice of comparators {6b}

Local wound infiltration with levobupivacaine has been the most commonly used mode of postoperative analgesia in laparoscopic surgery in our clinic. To explore the possibilities of improving the efficacy of postoperative pain management after peritoneal endometriosis surgery, we decided to choose LTAP with levobupivacaine as a comparator. Administration of LTAP has a fast learning curve and it does not increase the operative time markedly.

### Intervention description {11a}

#### Outpatient clinic

After giving informed consent, study participants will fill in preoperative validated questionnaires regarding pain (Numeric Rating Scale, NRS) and endometriosis-related health (Endometriosis Health Profile, EHP-30).

#### Operating room; anesthesia

Total intravenous anesthesia will be administered to both groups in a standardized fashion using propofole and remifentanil infusions. No inhalation anesthetics will be used. Dexamethasone 5 mg iv will be administered for the prevention of postoperative nausea and vomiting (PONV).

#### Operating room; interventional treatment

Operating room (OR) nurse will randomize participants to intervention or control group with an allocation ratio of 1:1 by fetching a sealed opaque numbered envelope from the office of the operating ward. The envelope will be opened and sealed again and the levobupivacaine and saline will be prepared in identical looking syringes according to allocation before the operating surgeon enters the OR. The LTAP and local wound analgesia will both be administered at the beginning of the operation under laparoscopic vision by the surgeon. LTAP will be infiltrated bilaterally at the upper and lower quadrants between front axillary and mid-clavicular lines in the depth reaching the space between transverse abdominal and internal oblique muscles. Local wound infiltration will be administered to all trocar sites prior to incision. The study group will be given LTAP with 20 ml levobupivacaine 2.5 mg/ml bilaterally and local wound infiltration with saline 10 ml in total as placebo. The control group will be administered with saline 20 ml bilaterally as placebo to the sites corresponding LTAP and local wound infiltration with levobupivacaine 5 mg/ml 10 ml in total.

#### Operating room; surgical technique

Standard surgical care according to our protocol in laparoscopic surgery of suspected peritoneal endometriosis will be given. Peritoneal endometriosis implants will be resected using a monopolar hook. Bipolar energy instrument may be used to ensure hemostasis where needed.

#### Postoperative analgesia

At the end of the procedure, 50 mg of dexketoprofen iv and fentanyl 0.5 μg/kg iv will be administered as remifentanil infusion is discontinued. As the patient arrives in the recovery room, a PCA pump (Delta Legacy iv) will be started with oxycodone 3 mg/ml with 1.5 mg boluses maximum of 4 per hour. Iv oxycodone may be administered in addition to the PCA-pump as a rescue analgesia. Paracetamol 1 g × 3 po and ibuprofen 600 mg × 3 po are started. Early mobilization and other ERAS protocols will be implemented.

### Criteria for discontinuing or modifying allocated interventions {11b}

Not applicable. Allocation and allocated intervention are done minutes apart on anesthetized patients, so no need for modification is expected.

### Strategies to improve adherence to interventions {11c}

The preoperative data as well as the outcome measure data during the hospital stay will be collected by the hospital staff. The study participants that have not returned the 6-month questionnaires will be contacted by the investigators to improve the response rate using their contact details (address, phone number) that can routinely be found in the clinical patient notes in Finland.

### Relevant concomitant care permitted or prohibited during the trial {11d}

Treatment of the patient will be conducted by standard care protocols regardless of the trial participation.

### Provisions for post-trial care {30}

Ancillary and post-trial care including the care of possible complications will be given according to standard medical practice irrespective to the trial itself. Every patient receiving medical or surgical care in Finland is covered by the Finnish Patient Insurance Centre. Possible complications caused by the study interventions are compensated via the center.

### Outcomes {12}

Primary outcome is the overall 24 h postoperative opioid consumption compared between the study groups. The amount of oxycodone administered via the PCA-pump and possible rescue analgesics (oxycodone administered in addition to the PCA-pump) will be converted to morphine equivalents.

As secondary outcomes, the following will be measured:

1. Postoperative pain using NRS scaled from 0 to 10 (0 meaning no pain and 10 meaning “worst imaginable pain”). The maximum experienced pain will be recorded at the recovery room. Additional NRS will be recorded at the ward every six hours up to 24 h (if the patient is discharged before 24 h, they are requested to fill in the NRS at home).
2. Factors related to ERAS (nausea, vomiting, peroral intake, mobilization, complications, time of discharge, readmission to hospital) will serve as other outcomes and will be documented and analyzed accordingly.
3. A 6-month postoperative follow-up will be conducted using NRS pain inquiry and EHP-30 questionnaire being sent to participants.

### Participant timeline {13}

### Sample size {14}

Sample size was calculated based on the assumption that LTAP would decrease the postoperative opioid consumption by 50%, which was thought to be a clinically relevant difference. The calculation was based on previous literature showing a 15.4 mg ± 9.2 postoperative opioid consumption after local wound analgesia [15]. With 80% power and 0.05 alpha error, the sample size of 46 (23 + 23) was obtained to detect a decrease of 7.7 mg in opioid consumption from 15.4 mg in the control group to 7.7 mg in the intervention group, assuming a standard deviation of 9.2 mg. Sample size was calculated according to Chow et al. [16], and the calculation was performed with statistical program R.

### Recruitment {15}

All eligible patients referred to gynecological outpatient clinic at the Oulu University Hospital with diagnosed or suspected peritoneal endometriosis needing laparoscopic evaluation or surgery because of insufficient response to medical management will be considered as potential trial participants. After receiving thorough information on the study protocol including possible advantages and disadvantages, and after voluntary signing of the informed consent, the trial participants will be enrolled to the LTAP-trial.

## Assignment of interventions: allocation

### Sequence generation {16a}

A computer-created random allocation list with simple randomization will be created and numbered; sealed opaque letters will be done to confirm blinding. The list will be created by the study statistician and the envelopes by the study nurse who are not involved in data collection or patient care.

### Concealment mechanism {16b}

The allocation sequence will be concealed by using sequentially numbered, opaque sealed envelopes.

### Implementation {16c}

The sealed envelopes will be opened and concealed again in the operating room prior to the operation by the anesthetic nurse and the OR nurse. The nurses will not participate in the treatment of the patient outside the operating room. The enrollment will be performed by the surgeons at the pre-surgery visit at the outpatient clinic.

## Assignment of interventions: blinding

### Who will be blinded {17a}

Trial participants, surgeons, and the anesthesiologist will be blinded to interventions. All participants will be administered both LTAP and local wound injections (analgesic and placebo) as described earlier. The injectable analgesics will be prepared by the OR nurse and the anesthetic nurse, who also open the sealed envelopes. The needles, the syringes, and the injectable amounts are the same in both groups.

### Procedure for unblinding if needed {17b}

In case of a severe allergic reaction after levobupivacaine infiltration, unblinding will be performed in order to reveal the exact dosage of levobupivacaine administered.

## Data collection and management

### Plans for assessment and collection of outcomes {18a}

Data will be collected prospectively on an electronic SPSS-database designed for this study. Validated questionnaires (NRS and EHP-30) will be used at the baseline and for collecting outcome data concerning postoperative pain and 6-month follow-up. These are filled in on paper questionnaires by the patients and transferred into SPSS database by two authors who double-check each other’s work.

### Plans to promote participant retention and complete follow-up {18b}

Any trial participant lost to follow-up will be contacted in order to complete the 6-month follow-up.

### Data management {19}

All data will be handled with utmost care and confidentiality. Data will be stored electronically with passwords and any manual data will be stored behind locked doors in the department. Data entry is possible only for the authoring investigators.

### Confidentiality {27}

The unique personal ID codes given by the Finnish government at birth or immigration will be used to link data originated at different phases of the study. For analyses, only pseudonymized data will be handled, using ID codes generated for this study. A separate key file connecting the unique personal ID codes and the study ID codes will be created and stored in hospital server where only primary investigators (AT, SK) have access.

### Plans for collection, laboratory evaluation, and storage of biological specimens for genetic or molecular analysis in this trial/future use {33}

Not applicable. No biological specimens will be collected.

## Statistical methods

### Statistical methods for primary and secondary outcomes {20a}

Statistical analyses will be performed on an intention-to-treat basis using IBM SPSS software by the study statistician who does not participate in patient care or data collection. Continuous variables will be described as mean with standard deviation (SD) or as median with 25th–75th percentiles and categorical variables as numbers and percentage of proportions. Comparison between the study groups will be conducted using the Student’s *t*-test or Welch test for continuous variables, Mann-Whitney *U* test for variables measured at ordinal scale, and the chi-square or Fisher’s test for categorical variables. Furthermore, continuous variables with measurements both pre- and postoperatively will be analyzed using linear regression model with preoperative measurement as an adjusting factor. The statistical significance limit is set at two-sided *p*-value < 0.05.

### Interim analyses {21b}

No interim analyses will be performed.

### Methods for additional analyses (e.g., subgroup analyses) {20b}

No preplanned subgroup analyses will be performed.

### Methods in analysis to handle protocol non-adherence and any statistical methods to handle missing data {20c}

Per-protocol analyses will be performed as sensitivity analyses when protocol violations occur. In the case of missing data, a multiple imputation (MI) method will be used. If the results of the MI analysis differ from the original, then both results will be presented. In the case of an arbitrary missing case pattern, the fully conditional specification will be used as an imputation method. In the case of a monotone missing case pattern, the regression imputation will be used.

### Plans to give access to the full protocol, participant level-data, and statistical code {31c}

Health data is sensitive data and cannot be delivered even if pseudonymized. Statistical code and output may be presented if asked.

## Oversight and monitoring

### Composition of the coordinating center and trial steering committee {5d}

Not applicable. This study is a single-center clinical trial with a short follow-up time and low risks for the participating patients.

### Composition of the data monitoring committee, its role and reporting structure {21a}

No data monitoring committee will be needed for this single-center study.

### Adverse event reporting and harms {22}

Possible adverse events and other unintended effects of the trial will be documented on trial data and medical records. All significant adverse events will be listed specifically. Lethal or severe adverse events will be reported to the Finnish Medicines Agency (Fimea) as soon as possible or within 7 days from getting informed of the adverse event.

### Frequency and plans for auditing trial conduct {23}

All suspected severe adverse events and a statement regarding the safety of the trial participants will be reported to Fimea once a year. Any significant novel perceptions of the medicinal product will be reported to Fimea immediately.

### Plans for communicating important protocol amendments to relevant parties (e.g., trial participants, ethical committees) {25}

In case of possible future protocol modifications, The Ethical Committee at Oulu University Hospital as well as Fimea will be informed.

### Dissemination plans {31a}

The trial results will be published in international peer-reviewed journals focusing on the investigatory field in question.

## Discussion

The purpose of this study is to evaluate the efficacy and safety of laparoscopically guided transversus abdominis plane block (LTAP) in comparison to trocar site local analgesia in laparoscopic surgery for suspected or diagnosed superficial endometriosis. Previously, it has been shown that women with chronic pelvic pain or endometriosis have altered pain experience in form of lower pressure-pain threshold and lower maximal pain tolerance than controls, widespread myofascial dysfunction and pain sensitization beyond the pelvic area [17–19]. Additionally, severe dysmenorrhea, a leading symptom of endometriosis, has been noted to predict the severity of postoperative pain after gynecological laparoscopy [3]. Thus, there is a need for effective postoperative pain management regimen with minimal side effects allowing ERAS. So far, knowledge on LTAP in gynecologic surgery is sparse and inconsistent [12–14]. However, data from gastrointestinal surgery, mainly from laparoscopic cholecystectomies, have shown promising results after using LTAP in postoperative analgesia [7, 8, 11]. This study will offer knowledge on whether LTAP is an efficient tool for postoperative pain management for gynecological patients that would carry minimal risks and diminish postoperative opioid consumption as well as support enhanced recovery and discharge.

## Trial status

This is the protocol number one. The recruitment will begin in April 2021 and will be completed by the end of 2022.

## Abbreviations

ASA: Physical status classification system defined by American Society of Anesthesiologists
EHP-30: Endometriosis Health Profile -30
ERAS: Enhanced recovery after surgery
LTAP: Laparoscopically guided transversus abdominis plane block
NRS: Numeric Rating Scale
N/A: Not applicable
PCA: Patient-controlled analgesia pump
PONV: Postoperative nausea and vomiting
TAP: Transversus abdominis plane block
UTAP: Ultrasound-guided transversus abdominis plane block
